# COVID-19 masks: A barrier to facial and vocal information

**DOI:** 10.3389/fnins.2022.982899

**Published:** 2022-09-23

**Authors:** Nadia Aguillon-Hernandez, Renaud Jusiak, Marianne Latinus, Claire Wardak

**Affiliations:** UMR 1253, iBrain, Université de Tours, Inserm, Tours, France

**Keywords:** speech, emotion, face, voice, occlusion, age

## Abstract

With the COVID-19 pandemic, we have become used to wearing masks and have experienced how masks seem to impair emotion and speech recognition. While several studies have focused on facial emotion recognition by adding images of masks on photographs of emotional faces, we have created a video database with actors really wearing masks to test its effect in more ecological conditions. After validating the emotions displayed by the actors, we found that surgical mask impaired happiness and sadness recognition but not neutrality. Moreover, for happiness, this effect was specific to the mask and not to covering the lower part of the face, possibly due to a cognitive bias associated with the surgical mask. We also created videos with speech and tested the effect of mask on emotion and speech recognition when displayed in auditory, visual, or audiovisual modalities. In visual and audiovisual modalities, mask impaired happiness and sadness but improved neutrality recognition. Mask impaired the recognition of bilabial syllables regardless of modality. In addition, it altered speech recognition only in the audiovisual modality for participants above 70 years old. Overall, COVID-19 masks mainly impair emotion recognition, except for older participants for whom it also impacts speech recognition, probably because they rely more on visual information to compensate age-related hearing loss.

## Introduction

Faces and voices are primary vectors of information crucial for social interaction and communication. Since March 2020, due to the COVID-19 pandemic, the world is moving forward masked. To prevent virus spread, national health agencies in multiple countries recommend wearing a mask that covers the mouth and nose. While face covering occurs in normal situation depending on cultural contexts or environment, such as wearing a scarf, sunglasses, or a niqab, wearing a surgical mask gives the general impression of larger disruption of social interaction (Saunders et al., 2021). Faces are processed holistically (as a whole) rather than analytically (feature by feature; Maurer et al., 2002). Facial expressions have been shown to involve both analytical and holistic processing depending on the emotion (Meaux and Vuilleumier, 2016). Diagnostic features for a particular emotion are in specific parts of the face (Blais et al., 2012); for instance, while happiness recognition seems to rely more on the bottom part of the face, the recognition of sadness or fear appears to depend more on the eye region (Bombari et al., 2013). Face covering may therefore impact the type of processes involved in facial perception by hindering global information and making it more dependent on facial features; in addition, covering the bottom or top part of the face may have differential effect on emotion recognition. It thus appears crucial to compare the effect of wearing a mask to wearing other accessories that cover different parts of the face. A face mask also impedes the transmission of acoustical information from the voice (e.g., Nguyen et al., 2021) and hides articulatory movements of the mouth, important for lip reading and speech comprehension. Therefore, face masking can impact face and voice information processing, as well as the audiovisual integration of social information.

Recently, there was a surge in research about the effect of mask on facial information recognition and in particular emotions (Carbon, 2020; Fitousi et al., 2021; Gori et al., 2021; Grundmann et al., 2021; Kastendieck et al., 2021; Marini et al., 2021; Noyes et al., 2021; Pazhoohi et al., 2021), with many studies reporting impaired emotion and identity recognition in masked faces. Partial occlusion of the face (Fischer et al., 2012; Kret and de Gelder, 2012a) is known to disrupt more the recognition of positive emotions than negative ones. Consistently, a surgical mask appears to impact more the recognition of happiness than negative emotions (Fitousi et al., 2021; Marini et al., 2021; Grenville and Dwyer, 2022; Levitan et al., 2022; Ross and George, 2022), except sadness for which mixed results are reported (e.g., Marini et al., 2021; Grenville and Dwyer, 2022; Ross and George, 2022). In most studies, authors artificially added masks on still face photographs from existing face databases. Although allowing a comparison of controlled and identical emotional content between masked and non-masked conditions, these protocols using retouched images do not fully investigate ecological facial emotion recognition. First, really worn facial masks do not completely mask important structural information (Fitousi et al., 2021); note that no differences between real-worn and artificially added mask on still photographs were observed (Grenville and Dwyer, 2022). Second, facial expressions are better recognized on dynamic stimuli, especially when they are subtle (Bould and Morris, 2008). Consistently, physiological reactivity, reflecting automatic face processing, is sensitive to both the realism and the dynamism of a face (Aguillon-Hernandez et al., 2020), recommending the use of videos over photographs to study emotion perception. Therefore, using videos of persons really wearing masks appears more optimal and ecological to study the impact of face masking on facial emotion recognition. In addition, as has been done in a few studies (Roberson et al., 2012; Noyes et al., 2021), the use of other elements to mask the face (sunglasses or scarf) allows better control of the specific impact of the COVID-19 mask. Comparing different accessories (mask or scarf) to occlude the bottom half of the face might reveal a supplementary hindrance of the mask, possibly due to its negative psychological value (Saunders et al., 2021).

Using videos allows to investigate the effects of masks on audiovisual perception of both emotion and speech. This is important as in ecological context, faces are rarely seen in isolation and are often coupled with other cues. Audiovisual integration is particularly important in situation where the signal in one modality is degraded (de Boer et al., 2021), as is the case when the face is masked, stressing the importance of testing recognition with audiovisual stimuli. Adding information, for example, body cues in a purely visual context, has been shown to decrease the impact of face masks on emotion recognition (Ross and George, 2022).

This study aimed to measure the effect of wearing a surgical mask on the recognition of (1) visual facial emotion, with respect to other face covering accessories; (2) emotion; and (3) speech in voices, faces, and audiovisual stimuli, using a video database developed exclusively for the study. We hypothesized that face covering will impact facial emotion recognition, with differential effects for covering the bottom or top part of the face, and possibly a larger effect for mask. In the audiovisual emotion recognition task, we expected better emotion recognition in audiovisual than in visual-only condition as the auditory input would compensate for masking the mouth. In the audiovisual speech recognition task, we thought the mask could interfere with syllable recognition as the mask is known to alter transmission of acoustical information.

## Materials and methods

### Stimuli

**FIGURE 1:**
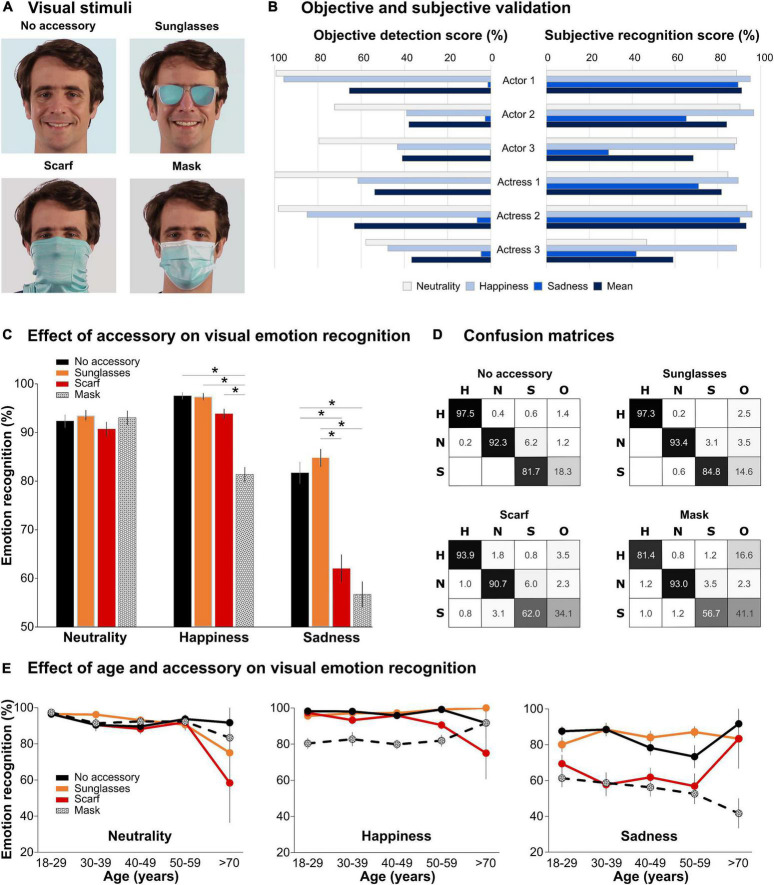
Effect of facial accessory on visual emotion recognition. **(A)** Visual stimuli. The four images depict the four accessory conditions (no accessory/sunglasses/scarf/mask) for one actor and one emotion (happiness). The images were extracted from the middle of the video when the emotion was expressed. The color of the background was adjusted, so that the four conditions had the same overall luminosity and colorimetry. **(B)** Objective and subjective validation. Objective detection scores correspond to FaceReader emotion probability converted in % for the six actors and the three emotions in the no accessory condition. Subjective recognition scores correspond to the mean emotion recognition for the same 18 videos by 124 participants. Videos from Actor 3 and Actress 3 were subsequently removed from the analyses, to evaluate the impact of an accessory only on emotions already correctly categorized. Neutrality is represented in light gray, happiness in light blue, sadness in medium blue, and the mean of the three emotions in dark blue. **(C)** Effect of accessory on visual emotion recognition. Histograms represent the mean recognition score (in %, ±standard error) for neutrality (left), happiness (middle), and sadness (right), in the four accessory conditions (black: no accessory, orange: sunglasses, red: scarf, and black-and-white pattern: mask). *p < 0.05. **(D)** Confusion matrices. For each accessory condition, the table presents the mean score (in %) of Happiness (H), Neutral (N), Sadness (S), or Other (O) responses (columns) as a function of the actual emotion in the video (lines: H/N/S). The gray level of each cell is proportional to the score (100%: black and 0%: white). **(E)** Effect of age and accessory on visual emotion recognition. Mean recognition score (in %, ±standard error) for neutrality (left), happiness (middle), and sadness (right), in the four accessory conditions (same color code as in panel **C**) for the five age groups.

**FIGURE 2:**
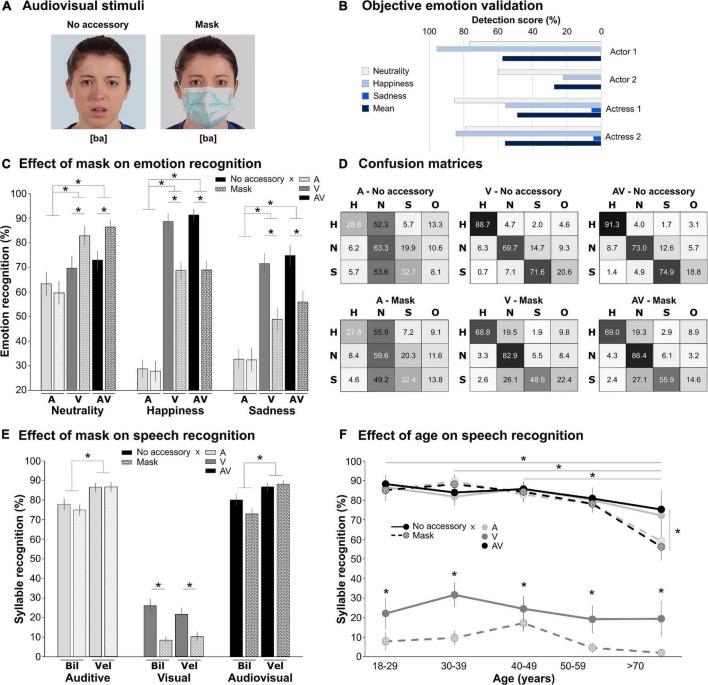
Effect of mask on audiovisual emotion and speech recognition. **(A)** Audiovisual stimuli. The two images depict the two accessory conditions (no accessory/mask) for one actress, one emotion (sadness), and one syllable ([ba]). The images were extracted from the middle of the video when the emotion was expressed and the syllable articulated. The color of the background was adjusted, so that the two conditions had the same overall luminosity and colorimetry. **(B)** Objective emotion validation. Detection scores correspond to FaceReader emotion probability converted in % for the four actors and the three emotions in the no accessory condition (pooled across syllables). **(C)** Effect of mask on emotion recognition. Histograms represent the mean recognition score (in %, ±standard error) for neutrality (left), happiness (middle), and sadness (right), in the three modalities (A: auditive, light gray; V: visual, middle gray; and AV: audiovisual, black) in the two accessory conditions (no accessory: full, mask: pattern). *p < 0.05. **(D)** Confusion matrices. For each accessory × modality (A/V/AV) condition, the table presents the mean score (in %) of Happiness (H), Neutral (N), Sadness (S), or Other (O) responses (columns) as a function of the actual emotion in the video (lines: H/N/S). The gray level of each cell is proportional to the score (100%: black, 0%: white). **(E)** Effect of mask on speech recognition. Histograms represent the mean recognition score (in %, ± standard error) for auditive (left), visual (middle), and audiovisual (right) modalities, for the bilabial (Bil) and velar (Vel) syllables in the two accessory conditions (same color code as in panel **C**). *p < 0.05. **(F)** Effect of age on speech recognition. Syllable recognition score (in %, ±standard error) for the three modalities and the two accessory conditions (same color code as in panel **C**), pooled across syllables, as a function of the age group. *p < 0.05.

We created two sets of videos by filming six actors (three males), starting in a neutral state and either staying neutral or expressing emotions (happiness or sadness; chosen as they do not yield avoiding behavior) before returning to neutral. During filming, actors worn the accessories and thus expressed the emotions as the accessories allowed them to do, mimicking as much as possible real-life emotional expression, without exaggerating emotional intensity. For neutral expressions, actors stayed neutral and unmoving, but the small motion of the face muscles could be observed. Actors were told to produce the same emotions in all conditions. They had to observe a visual cue (without producing saccades) moving on a Gaussian curve to induce emotional expression with the same dynamic and intensity in each condition. In Set 1 (Figure 1A), they were silent and wore an accessory (sunglasses/scarf/mask) or not. A total of 72 videos were created: 6 actors × 3 emotions × 4 accessories. In Set 2 (Figure 2A), four of the previous actors were bare face or wore a surgical mask while articulating either bilabial ([pa]/[ba]) or velar ([ka]/[ga]) syllables and expressing or not an emotion. For each take, three versions were created by removing the visual information for the auditory-only version, the auditory information for the visual-only, and keeping them together for the audiovisual version. There were 288 videos: 4 actors × 3 modalities × 3 emotions × 4 syllables × 2 accessories. Videos were edited to last 2 s, frame each face identically, and were equated in luminosity and colorimetry (see Supplementary material). Soundtracks for each video were normalized in intensity and energy, so no difference in sound intensity was present between masked and unmasked utterances.

Videos without accessories were objectively validated for the intended emotion with FaceReader (FR6; Lewinski et al., 2014). FR6 determines the intensity (on a 0–1 scale) of each specific emotion by estimating the configuration of 20 facial action units (FAU) activated in the expression at each time frame. Intensity score allows emotion categorization. We report the emotion at the maximum of intensity for the intended emotion between 0.5 and 1.5 s, corresponding to the emotion-inducing visual cue. FR6 was calibrated on the neutral video of each actor; then, videos without accessories were analyzed. Objective validation was not possible for the partially occluded videos.

### Protocol

The study ran online,^1^ with four successive steps: (1) demographic questionnaires; collection of participant’s biological sex and age (five categories; 18–29, 30–39, 40–49, 50–69, and more than 70 years old); known developmental disorders (three choices: yes, no, “I don’t want to answer”); (2) visual emotion discrimination task; (3) autism quotient (AQ; Lepage et al., 2009; see Supplementary material); (4) audiovisual task. Participation was anonymous, and participants could stop at any time. The total duration of the study was about 30 min. This study was approved by the local ethical committee (CER-TP-2021-05-04).

In the visual emotion recognition task, participants were presented a four-alternative forced choice (AFC) (happy, neutral, sad, and other) after each of the 72 videos of Set 1. In the audiovisual task, each participant was shown 96 videos selected randomly from Set 2 (except 5 who saw all the videos). After each video, participants were presented a four-AFC for the emotion recognition (happy, neutral, sadness, and other) and a five-AFC for the syllable recognition (“Ba,” “Pa,” “Ga,” “Ka,” and “other”).

### Participants

Detailed information on the inclusion of participants can be found in Supplementary material. For subjective validation of stimuli without accessories, data from 124 participants who completed the task were used to ensure that the posed emotions were correctly recognized by the participants.

For each recognition task independently, participants with performance outside three standard deviations from the mean were considered outliers and were excluded from the analysis.

In the visual emotion recognition task, 133 participants who completed a minimum of 35 trials were considered for analysis. The final sample for statistical analysis included 122 (32 males, 90 females, Table 1) participants (11 outliers). These participants provided enough data to obtain a recognition score for each emotion × accessory category.

**TABLE 1:** Repartition of participants according to age and gender in the three tasks.

Final sample	18–29 years old	30–39 years old	40–49 years old	50–69 years old	>70 years old	Total
***Visual emotion discrimination task***						
Female	23	20	26	20	1	90
Male	5	6	10	9	2	32
Total	28	26	36	29	3	122
***Audiovisual emotion discrimination task***						
Female	6	6	8	5	2	27
Male	1	3	3	3	4	14
Total	7	9	11	8	6	41
***Audiovisual speech recognition task***						
Female	6	6	8	5	3	28
Male	1	3	3	4	2	13
Total	7	9	11	9	5	41

In the audiovisual task (which includes audio-only, visual-only, and audiovisual stimulation), 43 participants were included. In the audiovisual emotion recognition task, recognition scores were calculated for each emotion × accessory × modality category for 41 participants (two outliers; 14 males, 27 females; Table 1); average number of trials per condition was 7 [range: 1 16]. In the audiovisual speech recognition task, recognition scores were calculated for each syllable × accessory × modality category for 41 participants (two outliers; 13 males, 28 females; Table 1); average number of trials per condition was 10 [range: 1 24]. In the visual-only condition, participants were deemed to recognize speech through lip reading.

### Statistical analysis

Data were analyzed with a repeated measure ANOVA within the general linear model (GLM) framework using Statistica. Gender was never included due to the strong imbalance in our sample. First, as our population age range was large and age is known to have an emotion-dependent effect on emotion recognition (e.g., West et al., 2012), the GLM included age as a categorical factor (five levels) to make sure data could be pooled across age range. If no effect was found, a final model was run without it. Greenhouse–Geisser correction was applied for data sphericity when needed, and analyses were completed with Bonferroni *post-hoc*.

In the visual emotion recognition task, the GLM included emotion (three levels: happiness, neutral, and sadness) and accessory (four levels: none, sunglasses, scarf, and mask) as within-subject factors. For the audiovisual emotion recognition task, the results were pooled across syllables, and the effects of emotion (three levels: happiness, neutral, and sadness), modality (three levels: audio, audiovisual, and visual), and accessory (two levels: none and surgical mask) were tested. For the audiovisual speech recognition task, the results were pooled across emotions and syllable types (velar vs. bilabial), and the effects of syllable type (two levels: bilabial and velar), modality (three levels: audio, audiovisual, and visual), and accessory (two levels: none and surgical mask) were tested.

## Results

### Visual emotion recognition task

#### Validation of the videos without accessory

Categorization (intensity scores converted in %) of neutral expression and happiness by FR6 was much higher than that of sadness (Figure 1B) for all actors. Emotion recognition for the 18 videos without accessories ranged from 29 to 96% (Figure 1B). Based on this validation, two actors were removed (Actor 3 and Actress 3 on Figure 1B) from subsequent analyses as their emotions were not well recognized. Sadness was poorly categorized by FR6, but categorization remained above chance for subjective validation. Emotions in the four actors selected were recognized by more than 60% of the participants, with sadness being less well recognized.

#### Effect of accessory on emotion recognition

GLM analysis with age as a categorical factor revealed a main effect of emotion [*F*(1.5,170.6) = 41.3, *p* < 0.0001], a main effect of accessory [*F*(3,351) = 37.6, *p* < 0.0001], an emotion by accessory interaction [*F*(4.9,567.6) = 12.4, *p* < 0.0001], and a three-way interaction [*F*(19.4,567.6) = 1.75, *p* = 0.015]. As can be observed in Figure 1C, sadness was overall less recognized than happiness or neutrality (*p* < 0.0001). The mask and the scarf significantly affected emotion recognition (*p* < 0.001 against the two remaining conditions), with worsened performance for the mask (*p* < 0.0001). Specifically, the mask interfered with happiness recognition (*p* < 0.0001) while the scarf did not (*p* > 0.9). Moreover, the mask and scarf interfered with sadness recognition (*p* < 0.0001) but did not differ. Age interacted with emotion and accessory (Figure 1E): Happiness recognition decreased with the mask, except for older participants, who were more affected by the scarf. The recognition of neutral expression was not affected by accessories and decreased with age, in particular for sunglasses and scarf. Sadness recognition was low for both scarf and mask in all age range except in older participants, who were affected by the mask but not the scarf.

Figure 1D shows the confusion matrices for the four accessory conditions. Overall, when participants did not correctly identify the emotion, they chose the response “Other,” as if they could not categorize what they saw or that they perceived another emotion not proposed in the choices (like anger or disgust). The neutral condition tended to be categorized more as “Sad” than “Other.” Masking the face, in happiness or sadness conditions, increased incertitude, as seen in the augmentation of “Other” responses, rather than making the faces seem more neutral.

### Audiovisual task

#### Objective validation of videos without accessory

The decoding scores of FR6 for the visual-only videos of Set 2 without accessory are presented in Figure 2B (data pooled across syllables). As was observed in the visual task, the categorization of neutral expression and happiness was much higher than that of sadness.

#### Effect of mask on emotion recognition

When age was included in the GLM, there was an effect of age [*F*(4,36) = 2.69, *p* = 0.046], due to an overall decrease in accuracy with increasing age, but no interaction with other factors. The GLM without age revealed significant main effects of emotion [*F*(2,72) = 21.5, *p* < 0.0001], modality [*F*(1.4,49.4) = 115.8, *p* < 0.0001], accessory [*F*(1,36) = 31.7, *p* < 0.0001], and interactions: (i) emotion by modality [*F*(3.1,111.6) = 12.54, *p* < 0.0001]; (ii) emotion by accessory [*F*(2,72) = 19.8, *p* < 0.0001]; (iii) modality by accessory [*F*(1.7,61.6) = 3.8, *p* = 0.034]; and (iv) three-way interaction [*F*(4,144) = 9.06, *p* < 0.0001]. As can be observed in Figure 2C, accuracy was better for neutral than happiness and sadness recognition, which also differed (*p* < 0.01 for each comparison). Emotion recognition was worse in the auditory modality (*p* < 0.0001 for each comparison) than in the visual and audiovisual conditions which did not differ. Emotion of masked faces was less well recognized than that of non-masked faces, in particular for happiness and sadness (*p* < 0.001). Mask had no effect on the recognition of neutral expression. The emotion by modality interaction revealed that while recognition for happiness and sadness was worse than for neutrality in the auditory modality (*p* < 0.0001 for each comparison), only sadness exhibited worse performances for the visual and audiovisual modalities (*p* < 0.02 for each comparison). For the three-factor interactions, we planned 12 comparisons: We tested the effect of the mask on the nine conditions, and we compared the performance for the visual and audiovisual masked conditions (with the hypothesis that the mask should have less deleterious effect in the audiovisual condition as the auditory input could compensate for masking the mouth). Face mask altered recognition of happiness and sadness (*p* < 0.0001), but improved recognition of neutral expression (*p* < 0.002), in visual and audiovisual conditions. Masking had no effect on emotion recognition in the auditory modality. There was no difference between visual and audiovisual conditions for masked faces.

Figure 2D shows the confusion matrices for the three modalities and two accessories. In the auditory modality, there was a general bias toward the “Neutral” choice, suggesting that syllables did not convey emotional content. In the visual and audiovisual modalities, the mask biased the responses of happiness and sadness toward the “Neutral” choice (whereas it was biased toward “Other” in the visual experiment 3.1.2).

#### Effect of mask on speech recognition

A GLM with age as a categorical factor revealed main effects of age [*F*(4,36) = 7.37, *p* < 0.001], syllable type [*F*(1,36) = 18.8, *p* < 0.001], modality [*F*(1.4,50.4) = 682.8, *p* < 0.001], accessory [*F*(1,36) = 23.5, *p* < 0.001], and interactions between syllables and accessory [*F*(1,36) = 10.01, *p* = 0.003], syllables and modality [*F*(2,72) = 9.86, *p* < 0.001], and modality and accessory [*F*(2,72) = 12.5, *p* < 0.001]. This later interaction was further characterized by an interaction with age [*F*(8,72) = 2.1, *p* = 0.047]. The main effect of age was driven by participants older than 70 who had syllable recognition accuracy inferior to all others age range (*p* < 0.01), except the 50–69 years old (*p* = 0.12).

Syllable recognition was overall better for velar syllables ([ga]/[ka]), in auditory and audiovisual modalities. Mask impaired syllable recognition (Figure 2E), an effect driven by the visual-only condition (*p* < 0.001), due to the absence of any indices to perform the task in visual-only condition with the mask. The syllables by accessory interaction showed that mask only affected the recognition of bilabial syllables (*p* < 0.001; for velar, *p* = 0.09). The syllables by modality interaction arose from velar syllables being better recognized than bilabial syllables in auditory and audiovisual modalities (*p* < 0.001) but not in the visual modality where performance did not differ (*p* > 0.5). To better comprehend the modality by accessory by age interaction, planned comparisons were performed to evaluate the effect of the mask for each modality and each age category (15 comparisons). These showed that while mask had an effect only on the recognition of visual-only syllables in adults below 70, in adults above 70 it also affected the recognition of syllables in the audiovisual condition (*p* = 0.009; *p* > 0.77 for all other age groups) (Figure 2F).

## Discussion

To study the impact of real-worn masks on social interaction, we recorded videos of actors expressing emotions with natural intensity, with and without real-worn accessories, to create more ecological audiovisual stimuli. Stimuli validation on bare faces showed that sadness was less well recognized than happiness, consistent with other studies (e.g., Carbon, 2020; Noyes et al., 2021). A worsened recognition of sadness compared to happiness could be related to sadness being driven by more subtle cues when actors are asked to perform natural emotions without exaggeration. The results showed an impact of surgical mask on emotion recognition, both in visual only and audiovisual settings, and on syllables perception. The first experiment showed that the effect of surgical mask was specific rather than due to the partial occlusion of the face.

Face masks have emotion-dependent effects on emotion recognition, with impairments observed for happiness (Marini et al., 2021; Grenville and Dwyer, 2022; Levitan et al., 2022), but not for anger or fear (Levitan et al., 2022; Ross and George, 2022). The effects of mask wearing on happiness recognition are relatively consistent across studies whether authors used still photographs of faces or whole bodies (Carbon, 2020; Kastendieck et al., 2021; Marini et al., 2021; Noyes et al., 2021; Grenville and Dwyer, 2022; Levitan et al., 2022; Ross and George, 2022), consistent with the bottom part of the face being more important in the perception of happiness. In agreement with these studies, but unlike Kastendieck et al. (2021) who used videos but with an artificially added mask, we found an effect of mask on happiness recognition. Consistent with our hypothesis, sunglasses did not affect happiness recognition (Noyes et al., 2021), however, neither did the scarf, possibly because it was very tight around the face so that one could see the raised cheeks. The mask-specific alteration of happiness recognition could be linked, not only to masking the mouth, but also to the negative bias of associating mask with infectious disease (Goh et al., 2020) that are not compensated by other information.

Cues important for sadness perception are mostly located in the upper part of the face (Bombari et al., 2013); yet, surprisingly, in our study, sadness recognition was more strongly impacted by wearing a face mask than happiness recognition. This result is both consistent (Carbon, 2020; Marini et al., 2021; Grenville and Dwyer, 2022) and inconsistent with previous studies (Ross and George, 2022). Lack of effect in the latter study possibly reflects compensation from other sources of information, such as body language. In addition, inconsistent with our hypothesis, we had no effect of sunglasses but an effect of scarf on sadness recognition, highlighting that with dynamic stimuli masking the bottom part of the face had a stronger impact on sadness recognition than masking the top part of the face. This could suggest that diagnostic features for faces differ between dynamic and static faces. It would be interesting to compare static and dynamic presentation of masked and non-masked faces. Note that although the mask impaired emotion perception, emotion recognition remained well above chance in our study which could reflect our choice to use an alternative forced-choice task with limited choices. Looking at the confusion matrices, it seems that incorrect answers were due to another emotion being perceived and could suggest that emotion recognition would be more impaired in a free-choice emotion recognition task.

In the audiovisual task, we reported a similar deleterious effect of mask on emotion recognition except for neutral expression, which was improved, due to a response bias toward the neutral choice. Audiovisual stimuli were used to assess whether non-facial information would help in recognizing emotion, as previously shown with body (Ross and George, 2022). However, in our videos, vocal information was not sufficient to compensate for the lost information, possibly because vocal emotions were very poorly recognized.

Perception of speech was affected by mask in particular for the perception of bilabial syllables regardless of modality, suggesting that wearing a mask impairs the production of syllables involving the lips, consistent with reports that face mask alters speech articulatory movements (Gama et al., 2021). However, we did not find a general impact of masks on intelligibility, contrary to Cohn et al. (2021) who reported altered perception for casual and positive speech, possibly due to controlling for intensity across masked and unmasked condition (Gama et al., 2021). In addition, for older participants, the mask impairs the recognition of syllables in the audiovisual condition, suggesting that they rely more on visual cues possibly to compensate potential hearing loss.

Overall, contrary to our expectations, audiovisual presentation did not improve recognition. This could be explained by the major weights of the visual and auditory information for evaluating emotional and speech contents, respectively, and the relatively weak information carried by the non-dominant modality. Conditions were therefore not optimal to produce audiovisual integration and observe a potential compensation of the complementary modality on masked-face perception.

Although our study was the first to test emotion and speech recognition on masked faces in dynamic stimuli, it has several limitations that should be resolved in future study. The study was run online to recruit a representative sample of the general population; however, due to the length of the study and the number of videos to be uploaded, numerous participants stopped the experiment before completing the study. Our sample is therefore inferior to what was expected. There was a large bias toward the participation of female in our study, which could have biased our results as female tends to perform better than male in emotion recognition tasks (Collignon et al., 2010; Kret and De Gelder, 2012b; Lambrecht et al., 2014; Abbruzzese et al., 2019). The audiovisual stimuli and the real-worn mask allowed us to be in a more ecological environment; nonetheless, faces and voices are rarely presented as were done here and often other cues are present at the same time, suggesting that in real life, other information may help deciphering the emotion expressed by individual. Nonetheless, the results for speech perception remain important and demonstrate that masking the face as a stronger impact on older people.

## Conclusion

Using a new controlled ecological audiovisual video database, we demonstrated that real-worn masks impact social interaction, including both emotion and speech perception and that this effect reflects a physical effect due to loosing information, and possibly a cognitive bias, due to the COVID-19 pandemic. Nonetheless, recognition of speech and emotion remained well above chance, suggesting the effect is less pronounced than originally thought.

## Data availability statement

The raw data supporting the conclusions of this article will be made available by the authors, without undue reservation.

## Ethics statement

The studies involving human participants were reviewed and approved by Comité d’Éthique pour la Recherche sur la Personne, Université de Tours et de Poitiers (Agreement CER-TP-2021-05-04). Written informed consent for participation was not required for this study in accordance with the national legislation and the institutional requirements. Written informed consent was obtained from the individual(s) for the publication of any potentially identifiable images or data included in this article.

## Author contributions

RJ created the database, including filming and editing the videos. NA-H, ML, and CW contributed equally to all other aspects of the study. All authors contributed to the article and approved the submitted version.

## Footnotes

1
https://pavlovia.org/
