# Supplementary material for: COVID-19 masks: A barrier to facial and vocal information
Source: Front Neurosci. 2022 Sep 23;16:982899. doi: 10.3389/fnins.2022.982899 (PMC9540850; doi:10.3389/fnins.2022.982899)
Supplement: Supplementary file 1 [file Data_Sheet_1.pdf]

Supplementary Material

Harmonization of light and chromatic features of videos

All videos were filmed in the same condition (light, framing, depth of field) in the professional studio of the digital laboratory of the University of Tours (Fac'LAb). All videos were post-processed with DaVinci Resolve 18® to last 2 s, to frame each face identically, and to be matched in colorimetry. Colorimetry of the videos (resolution: 1920*1080 pixel) was controlled by adjusting the RGB channels of all frames of each video in order to have the same intensity of red (mean = 0.7 ± 0.2), green (mean = 0.6 ± 0.2) and blue (mean = 0.7 ± 0.2) pixels. At the end, all frames of each video presented a constant luminosity of 25 Lux.

Participants

190 persons, above 18 years old, participated to this study. However, due to the number of videos to be loaded, participants' computers and internet networks performance could be a limitation and compromise reaching the end of the study; therefore, several participants did not complete all tasks. All answers were recorded even if the participant did not finish the study, explaining the variability in participants' numbers across tasks. From that initial sample, 36 were excluded because they stopped just after completing the demographic questionnaires.

The final sample comprised 154 participants (42 males; 112 females) with enough data to analyze emotion discrimination in the visual task. Four participants reported having neurodevelopmental disorders (two had dyslexia, one had Attention Deficit/Hyperactivity Disorder, and one had Autism Spectrum Disorder); two participants chose not to answer. These participants were included in the total sample.

Testing the effect of Autism Quotient

It has been proposed that individuals with Autism Spectrum Disorders (ASD) process faces less holistically than Typically-Developed (TD) individuals (e.g. Tanaka et al., 2012; Teunisse & de Gelder, 2003), or that their attentional bias away from the eyes affects the differential processing of the eyes or mouth regions (e.g. Rutherford et al., 2007; Tanaka et al., 2012). As a result, in the context of emotion recognition, they might be either less affected by mask wearing than their TD peers because they rely less on holistic processes, or more affected because they do not easily orient towards the eye region, the only remaining source of emotional cues. Pazhoohi et al. (2021) tested the influence of Autism Quotient (AQ) score on emotion recognition in masked faces. They showed that individuals with higher AQ scores showed an even more marked deficit in emotion recognition of masked faces that individuals with low AQ scores.

We took the opportunity of our study to confirm their results, but with a more detailed AQ estimation (AQ-50 instead of AQ-10).

Participants filled the AQ questionnaire in-between the purely Visual emotion discrimination task and the Audiovisual emotion/syllable recognition task. As a result of this design, we obtained AQ scores for 156 participants, with scores ranging from 4 to 37 (mean AQ score 16 ± 7 SD).

We included AQ as a continuous predictor in the GLMs, and when no effect was found GLMs were tested without it.

For the Visual emotion discrimination task (4.1.2 in the main Manuscript), AQ had no effect on emotion recognition accuracy, and did not interact with other factors.

For the Audiovisual emotion discrimination task (4.2.2 in the main Manuscript), AQ had no effect on emotion recognition accuracy, and did not interact with other factors.

For the Audiovisual syllable recognition task (4.2.3 in the main Manuscript), AQ had no effect on speech recognition accuracy, and did not interact with other factors.

In conclusion, AQ did not modulate perception in our study, unlike in Pazhoohi et al. (2021), possibly because our participants did not exhibit high scores (only 3 participants > 32).

Supplementary References

Pazhoohi, F., Forby, L., & Kingstone, A. (2021). Facial masks affect emotion recognition in the general population and individuals with autistic traits. *PloS One*, *16*(9), e0257740. https://doi.org/10.1371/journal.pone.0257740

Rutherford, M. D., Clements, K. A., & Sekuler, A. B. (2007). Differences in discrimination of eye and mouth displacement in autism spectrum disorders. *Vision Research*, *47*(15), 2099-2110. https://doi.org/10.1016/j.visres.2007.01.029

Tanaka, J. W., Wolf, J. M., Klaiman, C., Koenig, K., Cockburn, J., Herlihy, L., Brown, C., Stahl, S. S., South, M., McPartland, J. C., Kaiser, M. D., & Schultz, R. T. (2012). The perception and identification of facial emotions in individuals with autism spectrum disorders using the Let's Face It ! Emotion Skills Battery. *Journal of Child Psychology and Psychiatry, and Allied Disciplines*, *53*(12), 1259-1267. https://doi.org/10.1111/j.1469-7610.2012.02571.x

Teunisse, J.-P., & de Gelder, B. (2003). Face processing in adolescents with autistic disorder : The inversion and composite effects. *Brain and Cognition*, *52*(3), 285-294. https://doi.org/10.1016/s0278-2626(03)00042-3
